# Identification of novel non-synonymous variants associated with type 2 diabetes-related metabolites in Korean population

**DOI:** 10.1042/BSR20190078

**Published:** 2019-10-21

**Authors:** Tae-Joon Park, Heun-Sik Lee, Young Jin Kim, Bong-Jo Kim

**Affiliations:** Division of Genome Research, Center for Genome Science, Korea National Institute of Health, Chungcheongbuk-do 28159, Republic of Korea

**Keywords:** Exome array, Metabolite, Non-synonymous variant, Type 2 diabetes

## Abstract

Metabolome-genome wide association studies (mGWASs) are useful for understanding the genetic regulation of metabolites in complex diseases, including type 2 diabetes (T2D). Numerous genetic variants associated with T2D-related metabolites have been identified in previous mGWASs; however, these analyses seem to have difficulty in detecting the genetic variants with functional effects. An exome array focussed on potentially functional variants is an alternative platform to obtain insight into the genetics of biochemical conversion processes. In the present study, we performed an mGWAS using 27,140 non-synonymous variants included in the Illumina HumanExome BeadChip and nine T2D-related metabolites identified by a targetted metabolomics approach to evaluate 2,338 Korean individuals from the Korea Association REsource (KARE) cohort. A linear regression analysis controlling for age, sex, BMI, and T2D status as covariates was performed to identify novel non-synonymous variants associated with T2D-related metabolites. We found significant associations between glycine and *CPS1* (rs1047883) and PC ae C36:0 and *CYP4F2* (rs2108622) variants (*P<*2.05 × 10^−7^, after the Bonferroni correction for multiple testing). One of the two significantly associated variants, rs1047883 was newly identified whereas rs2108622 had been previously reported to be associated with T2D-related traits. These findings expand our understanding of the genetic determinants of T2D-related metabolites and provide a basis for further functional validation.

## Introduction

Metabolome-genome wide association studies (mGWASs) are useful for evaluating associations between genetic variants and serum or plasma metabolite levels by integrating genomics and metabolomics data [1]. Metabolites are thought to play important roles as markers and/or effectors of metabolic diseases or related traits, such as type 2 diabetes (T2D), hypertension, lipid levels, fasting plasma glucose, and fasting insulin levels. Thus, mGWASs are mainly aimed at refining and expanding our understanding of the causal determinants of metabolic diseases [2]. Several previous mGWASs using European cohorts from the Cooperative Health Research in the Region of Augsburg (KORA), Framingham Heart Study (FHS), and Twin UK have identified common variants in dozens of genetic loci associated with metabolite concentrations, including lipids, carbohydrates, amino acids, nucleotides, peptides, and cofactors [3–6]. In addition, ten common variants associated with metabolites related to T2D development have been identified in the Korea Association Resource (KARE) cohort [7].

However, mGWASs using existing genotype arrays, like Affymetrix 6.0, are generally focussed on non-coding variants located in intronic or intergenic regions, and have difficulty in capturing potentially functional genetic variants. To overcome this limitation, exome arrays focussed on potentially functional variants in protein-coding regions of genes are an alternative approach [8]. In particular, non-synonymous variants are critical for finding causal genetic factors for clinical phenotypes (e.g., metabolite changes) owing to their potential to alter protein function [8,9]. Recent exome-wide association studies using the Illumina HumanExome BeadChip have identified several associations between non-synonymous variants and metabolite traits. For instance, non-synonymous variants in five genes (*CTH, GMPS, HAL, PAH*, and *UPB1*) are associated with metabolite levels, including cystathionine, xanthosine, histidine, phenylalanine, and ureidopropionate, in European subjects from the FHS cohort, and these results have been replicated in the Atherosclerosis Risk in Communities cohort [8]. Additionally, associations of T2D-related metabolite ratios with three non-synonymous variants in *CHIA, REV3L*, and *APOE* have been identified [10].

In the present study, we searched for potentially functional genetic factors affecting the T2D risk based on associations with metabolite traits. Metabolite quantification by a targeted method and genotyping using the Illumina HumanExome BeadChip were used to evaluate 2,338 Korean subjects from the KARE cohort. Statistical analyses of genetic associations with T2D-related metabolites were performed. Non-synonymous variants located in two genes showed significant associations with T2D-related metabolites.

## Materials and methods

### Study subjects

The KARE cohort is a community-based cohort established through the Korean Genome Epidemiologic Study (KoGES) project. In the Ansung and Ansan areas of Kyounggi province, South Korea, 10,030 individuals were initially collected from 2001 to 2002 and six subsequent surveys were performed every 2 years from 2003 to 2014 [11]. Amongst the participants included in KARE survey 2 (from 2005 to 2006), 2338 individuals with both genomic (exome array) and metabolomic datasets were recruited for the present study. Individuals were classified into T2D (*n=* 512), prediabetes (PD, *n=* 862), and normal glucose tolerance (NGT, *n=* 964) groups according to the American Diabetes Association (ADA) diagnostic criteria based on fasting plasma glucose levels (FPG), glycated hemoglobin levels (hemoglobin A1c, HbA1c), and 2-h plasma glucose levels (2h-PG) [12].

### Genotyping and quality control

Genotype data were obtained from 2,338 Korean subjects in the KARE cohort using the Illumina HumanExome BeadChip v1.1. The genotypes were called by automated clustering and manual inspection using Illumina GenomeStudio v2011.1 according to the clustering method of the Cohorts for Heart and Aging Research in Genomic Epidemiology (CHARGE) consortium [13]. A total of 59,340 non-synonymous variants were selected from the genotype data to identify potentially functional variants which may have causal effects on T2D-related metabolite levels by altering the amino acid sequences of proteins. For genotype quality control, variants with minor allele frequency (MAF) <0.001 (0.1%), significant deviation from Hardy–Weinberg equilibrium (*P<*1.0 × 10^−6^), and a missing genotype rate over 5% were excluded. In total, 27,140 non-synonymous variants were included for the association analysis of T2D-related metabolites.

### Metabolite level assessment

Serum metabolites in the 2,338 subjects with genotyping data were quantitated by targetted metabolomics using the AbsoluteIDQ p180 Kit (Biocrates Life Sciences, Innsbruck, Austria), including 40 acylcarnitines, 21 amino acids, 19 biogenic amines, 15 sphingolipids, 90 glycerophospholipids, and one hexose. This kit enables the simultaneous quantification of metabolites by liquid chromatography and flow injection mass spectrometry. A sample of pooled serum from healthy controls was used as a reference standard and was quantitated 36 times in randomly selected positions on the kit to estimate reproducibility. To ensure data quality, each metabolite was required to meet the following three criteria: (1) the coefficient of variance for the metabolites in the reference standards was <25%; (2) 50% of the estimated metabolite concentrations in the reference standard was above the limit of detection, which was set to three times the median of the three blank samples within each kit; and (3) 50% of the estimated metabolite concentrations in the experimental samples was above the limit of detection. In total, 123 metabolites met the criteria, and the final metabolomics dataset contained 1 hexose), 12 acylcarnitines, 21 amino acids, seven biological amines, ten sphingomyelins, 32 diacyl (aa) phosphatidylcholines (PCs), 32 acyl-alkyl (ae) PCs, and eight lysoPCs. The concentrations of all analyzed metabolites are reported in units of μM.

## Statistical analyses

### Selection of T2D-related metabolites

The odds ratios (ORs) for single metabolites were calculated by logistic regression for comparisons of metabolite levels between two groups (NGT versus T2D and PD versus T2D) [7]. In the analyses, diabetic conditions and metabolite concentrations were considered as dependent and explanatory variables, respectively. For the linear regression analysis, β values were calculated based on the concentration of each metabolite as an explanatory variable and postprandial glucose was considered as a dependent variable. The cutoff for significance was determined according to Bonferroni correction for multiple testing (*P<* 4.07 × 10^−4^).

### Association between non-synonymous variants and T2D-related metabolites

**Figure 1:**
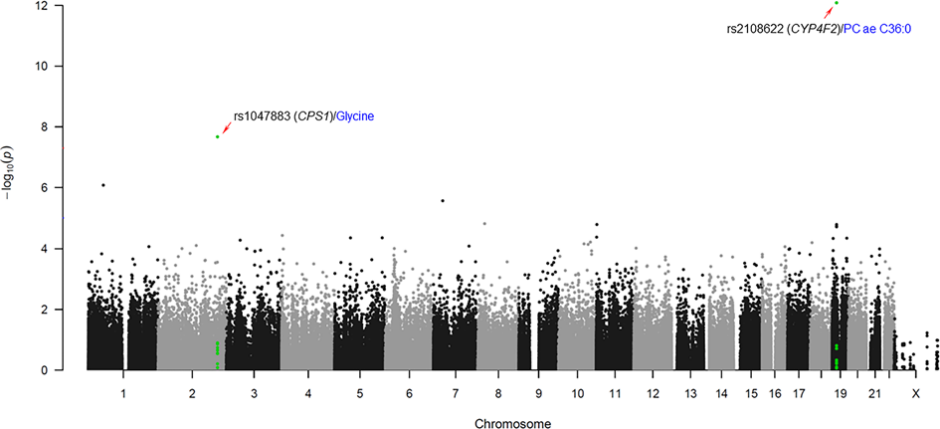
Manhattan plot of associations between T2D-related metabolites and non-synonymous variants Statistical significance of association indicated by negative logarithm of *P*-value (x-axis) is plotted against genomic position with the 22 autosomal chromosomes (y-axis). Chromosomal regions containing non-synonymous variants significantly associated with T2D-related metabolites are highlighted in green color.

Statistical analyses were performed to identify non-synonymous variants that are significantly associated with T2D-related metabolite levels. A linear regression analysis controlling for age, sex, body mass index (BMI), and T2D status (designated as a categorical predictor; normal = 1, PD = 2 and T2D = 3) as covariates was performed to evaluate the associations between allelic distributions of the 27,140 non-synonymous variants and nine T2D-related metabolite levels using PLINK (http://pngu.mgh.harvard.edu/∼purcell/plink). The concentration of each T2D-related metabolite was log transformed and normalized through inverse-rank method. Overall chromosomal positions and *P*-values for associations with T2D-metabolites for all non-synonymous variants were visualized by generating a Manhattan plot using R (version 3.6.0) (Figure 1). The threshold *P*-value for significance was adjusted by the Bonferroni correction for multiple testing (*P<* 2.05 × 10^−7^). Predictions of functional effects of the non-synonymous variants based on Sorting Intolerant from Tolerant (SIFT) and Polymorphism Phenotyping (Polyphen) software were obtained from the genome aggregation database (gnomAD, https://gnomad.broadinstitute.org).

## Results

### Characteristics of the study subjects

The clinical profiles of study subjects are summarized in Table 1. A total of 2,338 Korean individuals were divided into T2D (*n=*512), PD (*n=*862), and NGT (*n=*964) groups according to the ADA diagnostic criteria, as mentioned in the materials and methods. Parameters included in the clinical profiles differed significantly amongst the three groups (*P<*0.0001). Seven parameters (age, BMI, FPG, 2h-PG, HbA1c, total cholesterol [TCHL], and triglyceride [TG]) exhibited significantly higher values in the T2D group than in the NGT group, whereas the high-density lipoprotein (HDL) level was significantly lower in the T2D group (*P<*0.05). In addition, four parameters, i.e., FPG, 2h-PG, HbA1c, and TG, showed significant differences between the PD and T2D groups (*P<*0.05). These four parameters were higher in the T2D group than in the PD group.

**Table 1:** Clinical profiles of study subjects

Clinical profile	Total	NGT	PD	T2D	*P*-value^§^
Number of subjects (n)	2,338	964	862	512	
Age (yrs)*	57.1 ± 9.0	55.6 ± 8.9	58.0 ± 8.9	58.3 ± 8.9^†^	<0.0001
Sex (Male/Female)	1,121/1,217	426/538	415/447	280/232	
BMI (kg/m^2^)*	24.6 ± 3.2	23.5 ± 3.0	25.3 ± 3.2	25.5 ± 3.2^†^	<0.0001
FPG (mg/dl)*	95.6 ± 19.3	83.6 ± 5.2	98.3 ± 9.1	116.4 ± 29.8^†^ ^‡^	<0.0001
2h-PG (mg/dl)*	140.2 ± 64.3	92.2 ± 18.5	142.2 ± 31.8	239.1 ± 58.0^†^ ^‡^	<0.0001
HbA1c (%)*	5.7 ± 0.8	5.3 ± 0.3	5.7 ± 0.3	6.5 ± 1.1^†^ ^‡^	<0.0001
HDL (mg/dl)*	43.8 ± 10.2	45.3 ± 10.3	43.1 ± 9.8	42.1 ± 10.5^†^	<0.0001
TCHL (mg/dl)*	193.4 ± 36.2	186.6 ± 32.4	198.0 ± 38.9	198.5 ± 36.2^†^	<0.0001
TG (mg/dl)*	148.5 ± 118.4	114.3 ± 64.8	164.8 ± 144.6	185.3 ± 130.2^†^ ^‡^	<0.0001

* Values are indicated as mean ± standard deviation.

§ *P*-values were calculated using ANOVA evaluating the significant difference amongst the three groups.

† Values showing the difference between NGT and T2D (*P<*0.05).

‡ Values showing the difference between PD and T2D (*P<*0.05).

### T2D-related metabolites for association analysis

Amongst 123 quantified metabolites, nine were selected as T2D-related metabolites based on our previous study [7]. Metabolites showing significant differences in two pairwise comparisons (NGT and PD versus T2D) as well as the postprandial glucose level (*P<*4.07 × 10^−4^ after the Bonferroni correction for multiple testing) were selected (Table 2). The selected metabolites included two acylcarnitines (C14:1 and C16), one amino acid (glycine), one biogenic amine (creatinine), four glycerophospholipids (lysoPC a C18:2, PC aa C34:2, PC ae C36:0, and PC ae C36:2), and one sugar (hexose). Five metabolites, C14:1 (ORs = 1.62 and 1.57 for NGT and PD versus T2D, respectively), C16 (ORs = 1.73 and 1.40 for NGT and PD versus T2D, respectively), PC aa C34:2 (ORs = 1.58 and 1.31 for NGT and PD versus T2D, respectively), PC ae C36:0 (ORs = 1.43 and 1.57 for NGT and PD versus T2D, respectively), and hexose (ORs = 6.75 and 4.11 for NGT and PD versus T2D, respectively) were associated with an increased risk of T2D and a higher level of postprandial glucose (β values ranged from 0.11 to 0.46). In contrast, four metabolites, glycine (ORs = 0.45 and 0.69 for NGT and PD versus T2D, respectively), creatinine (ORs = 0.56 and 0.77 for NGT and PD versus T2D, respectively), lysoPC a C18:2 (ORs = 0.49 and 0.71 for NGT and PD versus T2D, respectively), and PC ae C36:2 (ORs = 0.64 and 0.77 for NGT and PD versus T2D, respectively), were negatively correlated with the risk of T2D and the postprandial glucose level (β values ranged from –0.25 to –0.12).

**Table 2:** T2D-related metabolites selected from previous report (Lee et al., 2016)

Metabolites*	NGT vs T2D		PD vs T2D		Postprandial glucose	
	OR (95% CI)	*P-*value	OR (95% CI)	*P-*value	Effect	*P-*value
C14:1	1.62 (1.42–1.84)	5.75E-13	1.57 (1.38-1.79)	3.99E-12	0.16	1.88E-14
C16	1.73 (1.51–1.98)	2.46E-15	1.40 (1.24–1.58)	7.68E-08	0.2	4.67E-21
Glycine	0.45 (0.39–0.52)	6.03E-27	0.69 (0.60–0.78)	1.07E-08	−0.23	1.69E-28
Creatinine	0.56 (0.48–0.65)	1.58E-13	0.77 (0.67–0.88)	1.27E-04	−0.12	5.48E-07
lysoPC a C18:2	0.49 (0.42–0.56)	1.78E-22	0.71 (0.63–0.81)	1.24E-07	−0.25	1.26E-31
PC aa C34:2	1.58 (1.39–1.81)	5.64E-12	1.31 (1.17–1.47)	4.00E-06	0.12	2.25E-08
PC ae C36:0	1.43 (1.26–1.63)	4.82E-08	1.57 (1.38–1.79)	4.02E-12	0.11	4.57E-07
PC ae C36:2	0.64 (0.56–0.73)	5.07E-11	0.77 (0.68–0.87)	3.80E-05	−0.14	3.43E-11
Hexose	6.75 (5.43–8.40)	9.14E-66	4.11 (3.41–4.95)	1.20E-49	0.46	1.19E-118

* C14:1, tetradecenoylcarnitine; C16, hexadecanoylcarnitine; lysoPC a C18:2, lysophosphatidylcholine acyl C18:2; PC aa C34:2, phosphatidylcholine diacyl C34:2; PC ae C36:0, phosphatidylcholine acyl-alkyl C36:0; PC ae C36:2, phosphatidylcholine acyl-alkyl C36:2

### Non-synonymous variants associated with T2D-related metabolites

A total of 27,140 non-synonymous variants were included in the association analysis for nine T2D-related metabolite levels to identify potentially functional variants, which may have causal effects after genotype quality control. A Manhattan plot summarizing the association analysis of all non-synonymous variants with respect to T2D-related metabolites is presented with chromosomal positions (x-axis) and the negative logarithm of *P*-values (y-axis) for each variant (Figure 1). We identified two non-synonymous variants that were significantly associated with T2D-related metabolites using a linear regression analysis controlling for age, sex, BMI, and T2D status as covariates (*P<*2.05 × 10^−7^, Table 3). These variants were located in the coding regions of two genes, *CPS1* (rs1047883) and *CYP4F2* (rs2108622). A common variant of the *CPS1* gene, rs1047883 (MAF = 0.470) was significantly associated with the glycine level (*P=*2.13 × 10^−8^). The A allele of rs1047883, inducing an amino acid change from alanine to threonine (Ala350Thr), was correlated with a decreased level of glycine (β = −0.155). A *CYP4F2* variant, rs2108622 (MAF = 0.325), was associated with PC ae C36:0 levels (*P=*8.19 × 10^−13^). The A allele of the variant causing a change in the amino acid sequence from valine to methionine (Val433Met) was associated with an increase in PC ae C36:0 levels (β = 0.213). Predictions of functional significance of the non-synonymous variants based on SIFT and Polyphen software were obtained from gnomAD. Results from both of the two predictions indicate that rs2108622 (predicted as ‘deleterious’ in SIFT and ‘probably damaging’ in Polyphen) may be a pathogenic variant (Table 3). In contrast, the rs1047883 was predicted to yield moderate effect (predicted as ‘tolerated’ in SIFT and ‘benign’ in Polyphen). The top five non-synonymous variants associated with the nine T2D-metabolites are listed in Supplementary Table S1.

**Table 3:** Significant associations between non-synonymous variants and T2D-related metabolites in KARE cohort (*P<*2.05 × 10^−7^)*

Metabolites^§^	SNP	Gene	Chr	Location	Alleles^†^	Amino acid change	MAF	β	*P*-value	Predicted functional effects	
										SIFT	Polyphen
Glycine	rs1047883	*CPS1*	2	211456637	G/A	Ala350Thr	0.470	−0.155	2.13E-08	Tolerated	Benign
PC ae C36:0	rs2108622	*CYP4F2*	19	15990431	G/A	Val433Met	0.325	0.213	8.19E-13	Deleterious	Probably damaging

* Threshold for significance has been adjusted for Bonferroni correction (*P<*0.05/[number of SNPs × number of T2D-related metabolites]).

† The allele of each non-synonymous variant is indicated as ‘major allele/minor allele’.

§ PC ae C36:0, phosphatidylcholine acyl-alkyl C36:0.

### Linkage disequilibrium status of non-synonymous variants associated with T2D-related metabolites

**Figure 2:**
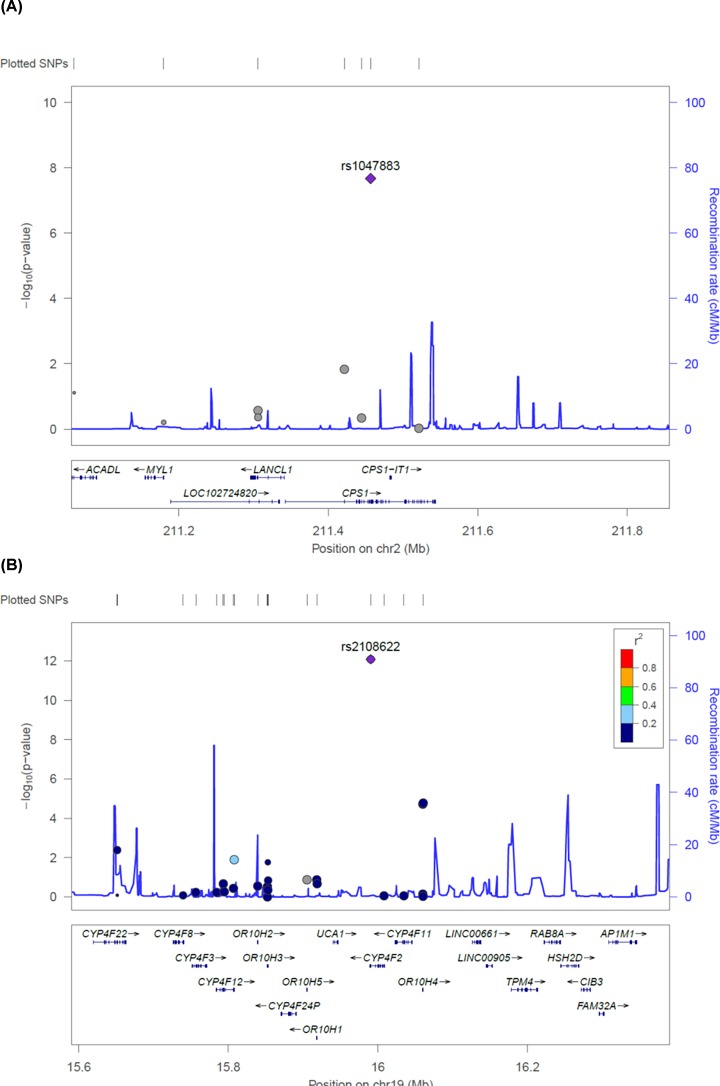
Regional plots for two significantly associated non-synonymous variants with T2D-related metabolites Variants are plotted by position on chromosome (x-axis) and log-scaled *P*-value (y-axis). The rs numbers for the most significant non-synonymous variants are shown on the plots. Recombination rates estimated based on 1000Genomes database are plotted in blue color to reflect local LD structure. The variants near the most significant markers are colored according to LDs between them (taken from pairwise *r^2^* values from the 1000Genomes Asian database). Genes, the position of exons and the direction of transcription are also plotted.

Regional plots for the two non-synonymous variants that were significantly associated with T2D-related metabolites are presented in Figure 2. Each variant is plotted according to chromosomal position (x-axis) and negative logarithm of the *P*-value (y-axis). Recombination rates in the flanking region of each variant, indicated as blue peaks in the figure, were predicted to reflect linkage disequilibrium (LD) structures based on the 1000 Genomes Asian database. No LD was observed in the flanking region of each variant, suggesting that the associations of these variants with T2D-related metabolites are independent of proxy variants.

### Relationship between metabolite levels and genotype in the NGT and diabetic groups

**Figure 3:**
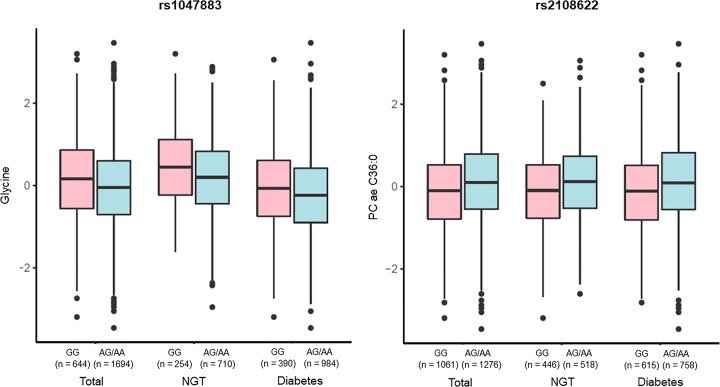
Relationship of metabolite levels to genotypes of two non-synonymous variants significantly associated with T2D-related metabolites in NGT and diabetic groups Box plots are visualizing the alteration of metabolite levels (y-axis) according to genotypes of the two non-synonymous variants associated with T2D-related metabolites (x-axis). The metabolite concentration values were log-transformed and normalized through inverse-rank method. In the boxplots, the top and bottom of boxes represent the 25th and 75th percentiles, respectively. Bold line in the middle of each box indicates the median.

Differences in metabolite levels according to genotype (carriers versus non-carriers of the minor allele) for each non-synonymous variant observed in overall subjects (‘total’), NGT, and diabetic (PD and T2D, ‘diabetes’) groups are summarized as boxplots in Figure 3. With respect to glycine levels, significantly higher values were observed in minor allele non-carriers (GG) than in minor allele carriers (AG and AA) for rs1047883 in all three groups (*P<*0.01). In contrast, PC ae C36:0 levels were observed significantly higher in minor allele carriers (AG and AA) than in minor allele non-carriers (GG) for rs2108622 (*P<*0.01). These results suggest that significant genotypic effects of the non-synonymous variants rs1047883 and rs2108622 on glycine and PC ae C36:0 levels exist.

## Discussion

We hypothesized that non-synonymous variants are more likely to include causal genetic factors associated with human disease-related phenotypes owing to their potential to alter protein function. Using linear regression, we investigated associations between non-synonymous variants captured in an exome array and nine T2D-related metabolites selected based on a previous study in the KARE cohort. As a result, two non-synonymous variants were associated with glycine and PC ae C36:0 levels, respectively.

A common non-synonymous variant located in the *CPS1* gene, rs1047883, was significantly associated with glycine levels in the present study. To the best of our knowledge, this variant has not yet been associated with glycine levels or T2D risk. The minor allele (A allele) of this variant, inducing an amino acid change from alanine to threonine (Ala350Thr), was related to a decrease in glycine levels. In Figure 3, significant genotypic effects related to a decrease in glycine levels were also observed in minor allele carriers (AG and AA). Considering that glycine levels decrease according to T2D progression, as mentioned in Table 2, the minor allele of rs1047883 may be associated with an increased risk of T2D. The effects of *CPS1* on glycine levels have been reported previously. *CPS1* encodes a mitochondrial carbamoyl-phosphate synthase that produces carbamoyl phosphate from ammonia and bicarbonate. Carbamoyl-phosphate synthase indirectly affects glycine levels by the interconversion between glycine and ammonia via the glycine cleavage complex [14]. Glycine is an amino acid involved in gluconeogenesis [15], and decreases in glycine levels are observed in diabetic rats and are associated with obesity and insulin resistance [16,17]. Several previous reports have suggested that *CPS1* variants are associated with glycine levels. For instance, a *CPS1* variant located in the 3′-untranslated region, rs715, is associated with glycine levels in T2D [14]. In addition, missense variants in the coding region of *CPS1* (rs1047891 and rs7422339) are associated with glycine levels [18,19]. Therefore, our results for the association between glycine and a *CPS1* variant are consistent with previous results.

A *CYP4F2* gene variant, rs2108622 was significantly associated with the PC ae C36:0 level. This is a known functional variant that reduces 20-hydroxyeicosatetraenoic acid (20-HETE) production, and is associated with hypertension, ischemic stroke [20,21], and metabolic syndrome, defined by the MetS score (sum of scores for waist circumference, blood pressure, lipid levels, and glucose levels), in the Swedish population [22]. The minor allele (A allele) of the rs2108622 polymorphism is associated with an increased level of PC ae C36:0. An amino acid substitution induced by rs2108622 was predicted to have a pathogenic effect using both SIFT and Polyphen (predicted as ‘deleterious’ and ‘probably damaging’, respectively) suggesting that this variant may affect PC ae C36:0 levels by alteration of CYP4F2 protein structure. Significant relationships between a decrease in PC ae C36:0 levels and the rs2108622 genotype were also observed for minor allele carriers (AG and AA) in Figure 3. Considering that the level of PC ae C36:0 increases according to T2D progression, as mentioned in Table 2, the minor allele of rs2108622 may be related to an increased risk of T2D. Functional roles of *CYP4F2* in T2D development have been reported previously. *CYP4F2* encodes a cytochrome P450 (CYP2) protein expressed in human pancreatic islets [23]. CYP4F2 induces the formation and release of 20-HETE, an eicosanoid metabolite of arachidonic acid mediated by CYP2-dependent ω-hydroxylase in human β-cells [24,25]. ω-Hydroxylase-mediated 20-HETE formation and release is stimulated by glucose, and glucose-stimulated 20-HETE formation and insulin secretion are reduced in T2D human islets [23]. Taken together, rs2108622 may affect T2D development by disrupting the function of CYP4F2 in 20-HETE formation in human β-cells.

The present study has some limitations. For instance, the observed associations between non-synonymous variants and T2D-related metabolites were not replicated in other cohorts. Furthermore, it is insufficient to assure the causality of these non-synonymous variants because there is a possibility that association signals of the variants are due to the LDs with causal non-coding variants not being included in exome array. Therefore, further investigations including fine-mapping with GWAS data are needed to validate our results. Nevertheless, we were able to identify novel genetic factors involved in the T2D risk that have not been found in previous GWAS by the investigation of associations with T2D-related metabolites.

In conclusion, our genetic association analysis of T2D-related metabolites focussed on non-synonymous variants to identify causal markers of the T2D risk. Novel non-synonymous variants that are significantly associated with T2D-related metabolite levels were found. Although further investigations are needed to validate our results, these findings extend our understanding of the genetic determinants of T2D.

## Supplementary Material

Supplementary Table S1

## Abbreviations

CV: coefficient of variance
FHS: Framingham Heart Study
FPG: fasting plasma glucose level
KARE: Korea Association REsource
LD: linkage disequilibrium
MAF: minor allele frequency
mGWAS: metabolome-genome wide association study
NGT: normal glucose tolerance
OR: odds ratio
PC: phosphatidylcholines
PD: prediabetes
SIFT: Sorting Intolerant from Tolerant
TCHL: total cholesterol
TG: triglyceride
T2D: type 2 diabetes

## References

[1] Rhee E.P., Ho J.E., Chen M.H., Shen D., Cheng S., Larson M.G. et al. (2013) A genome-wide association study of the human metabolome in a community-based cohort. Cell Metab. 18, 130–143 10.1016/j.cmet.2013.06.013 23823483 PMC3973158

[2] Suhre K., Shin S.Y., Petersen A.K., Mohney R.P., Meredith D., Wagele B. et al. (2011) Human metabolic individuality in biomedical and pharmaceutical research. Nature 477, 54–60 10.1038/nature10354 21886157 PMC3832838

[3] Demirkan A., van Duijn C.M., Ugocsai P., Isaacs A., Pramstaller P.P., Liebisch G. et al. (2012) Genome-wide association study identifies novel loci associated with circulating phospho- and sphingolipid concentrations. PLoS Genet. 8, e1002490 10.1371/journal.pgen.1002490 22359512 PMC3280968

[4] Illig T., Gieger C., Zhai G., Romisch-Margl W., Wang-Sattler R., Prehn C. et al. (2010) A genome-wide perspective of genetic variation in human metabolism. Nat. Genet. 42, 137–141 10.1038/ng.507 20037589 PMC3773904

[5] Shin S.Y., Fauman E.B., Petersen A.K., Krumsiek J., Santos R., Huang J. et al. (2014) An atlas of genetic influences on human blood metabolites. Nat. Genet. 46, 543–550 10.1038/ng.2982 24816252 PMC4064254

[6] Draisma H.H.M., Pool R., Kobl M., Jansen R., Petersen A.K., Vaarhorst A.A.M. et al. (2015) Genome-wide association study identifies novel genetic variants contributing to variation in blood metabolite levels. Nat. Commun. 6, 7208 10.1038/ncomms8208 26068415 PMC4745136

[7] Lee H.S., Xu T., Lee Y., Kim N.H., Kim Y.J., Kim J.M. et al. (2016) Identification of putative biomarkers for type 2 diabetes using metabolomics in the Korea Association REsource (KARE) cohort. Metabolomics 12, 178 10.1007/s11306-016-1103-9

[8] Rhee E.P., Yang Q., Yu B., Liu X., Cheng S., Deik A. et al. (2016) An exome array study of the plasma metabolome. Nat. Commun. 7, 12360 10.1038/ncomms12360 27453504 PMC4962516

[9] Ng P.C.and Henikoff S. (2006) Predicting the effects of amino acid substitutions on protein function. Annu. Rev. Genomics Hum. Genet. 7, 61–80 10.1146/annurev.genom.7.080505.115630 16824020

[10] Jager S., Wahl S., Kroger J., Sharma S., Hoffmann P., Floegel A. et al. (2017) Genetic variants including markers from the exome chip and metabolite traits of type 2 diabetes. Sci. Rep. 7, 6037 10.1038/s41598-017-06158-3 28729637 PMC5519666

[11] Kim Y., Han B.G.and Ko G.E.S.g (2017) Cohort profile: The Korean Genome and Epidemiology Study (KoGES) Consortium. Int. J. Epidemiol. 46, e20 10.1093/ije/dyv316 27085081 PMC5837648

[12] American Diabetes, A (2018) 2. Classification and Diagnosis of Diabetes: Standards of Medical Care in Diabetes-2018. Diabetes Care 41, S13–S27 10.2337/dc18-S002 29222373

[13] Grove M.L., Yu B., Cochran B.J., Haritunians T., Bis J.C., Taylor K.D. et al. (2013) Best practices and joint calling of the HumanExome BeadChip: the CHARGE Consortium. PLoS ONE 8, e68095 10.1371/journal.pone.0068095 23874508 PMC3709915

[14] Xie W., Wood A.R., Lyssenko V., Weedon M.N., Knowles J.W., Alkayyali S. et al. (2013) Genetic variants associated with glycine metabolism and their role in insulin sensitivity and type 2 diabetes. Diabetes 62, 2141–2150 10.2337/db12-0876 23378610 PMC3661655

[15] Floegel A., Stefan N., Yu Z., Muhlenbruch K., Drogan D., Joost H.G. et al. (2013) Identification of serum metabolites associated with risk of type 2 diabetes using a targeted metabolomic approach. Diabetes 62, 639–648 10.2337/db12-0495 23043162 PMC3554384

[16] Wijekoon E.P., Skinner C., Brosnan M.E.and Brosnan J.T. (2004) Amino acid metabolism in the Zucker diabetic fatty rat: effects of insulin resistance and of type 2 diabetes. Can. J. Physiol. Pharmacol. 82, 506–514 10.1139/y04-067 15389298

[17] Lustgarten M.S., Price L.L., Phillips E.M.and Fielding R.A. (2013) Serum glycine is associated with regional body fat and insulin resistance in functionally-limited older adults. PLoS ONE 8, e84034 10.1371/journal.pone.0084034 24391874 PMC3877144

[18] Kettunen J., Demirkan A., Wurtz P., Draisma H.H., Haller T., Rawal R. et al. (2016) Genome-wide study for circulating metabolites identifies 62 loci and reveals novel systemic effects of LPA. Nat. Commun. 7, 11122 10.1038/ncomms11122 27005778 PMC4814583

[19] Yu B., Zheng Y., Alexander D., Morrison A.C., Coresh J.and Boerwinkle E. (2014) Genetic determinants influencing human serum metabolome Amongst African Americans. PLoS Genet. 10, e1004212 10.1371/journal.pgen.1004212 24625756 PMC3952826

[20] Ward N.C., Tsai I.J., Barden A., van Bockxmeer F.M., Puddey I.B., Hodgson J.M. et al. (2008) A single nucleotide polymorphism in the CYP4F2 but not CYP4A11 gene is associated with increased 20-HETE excretion and blood pressure. Hypertension 51, 1393–1398 10.1161/HYPERTENSIONAHA.107.104463 18391101

[21] Fava C., Montagnana M., Almgren P., Rosberg L., Lippi G., Hedblad B. et al. (2008) The V433M variant of the CYP4F2 is associated with ischemic stroke in male Swedes beyond its effect on blood pressure. Hypertension 52, 373–380 10.1161/HYPERTENSIONAHA.108.114199 18574070

[22] Fava C., Montagnana M., Danese E., Sjogren M., Almgren P., Guidi G.C. et al. (2012) The functional variant V433M of the CYP4F2 and the metabolic syndrome in Swedes. Prostaglandins Other Lipid Mediat. 98, 31–36 10.1016/j.prostaglandins.2012.03.001 22484021

[23] Tunaru S., Bonnavion R., Brandenburger I., Preussner J., Thomas D., Scholich K. et al. (2018) 20-HETE promotes glucose-stimulated insulin secretion in an autocrine manner through FFAR1. Nat. Commun. 9, 177 10.1038/s41467-017-02539-4 29330456 PMC5766607

[24] Kroetz D.L.and Xu F. (2005) Regulation and inhibition of arachidonic acid omega-hydroxylases and 20-HETE formation. Annu. Rev. Pharmacol. Toxicol. 45, 413–438 10.1146/annurev.pharmtox.45.120403.100045 15822183

[25] Williams J.M., Murphy S., Burke M.and Roman R.J. (2010) 20-hydroxyeicosatetraeonic acid: a new target for the treatment of hypertension. J. Cardiovasc. Pharmacol. 56, 336–344 10.1097/FJC.0b013e3181f04b1c 20930591 PMC2953733

