# Supplementary material for: Identification of novel non-synonymous variants associated with type 2 diabetes-related metabolites in Korean population
Source: Biosci Rep. 2019 Oct 21;39(10):BSR20190078. doi: 10.1042/BSR20190078 (PMC6822494; doi:10.1042/BSR20190078)
Supplement: Supplementary Table S1 [file BSR-2019-0078_supp.pdf]

Supplementary Table 1 Top 5 association between each T2D-related metabolite and non-synonymous variant in KARE cohort

Metabolites*	SNP	Gene	Chr	Location	Alleles†	Amino acid change	MAF	Beta	*P*-value	Predicted functional effects	
										SIFT	Polyphen
C14:1	rs11937432	*HAUS3*	4	2233709	A/G	Ile586Thr	0.045	0.289	3.64E-05	Tolerated	Benign
	rs56228802	*MAP3K1*	5	56177692	C/G	Val889Leu	0.007	-0.698	4.38E-05	Deleterious	Benign
	rs74352089	*CD163L1*	12	7528041	G/A	Ala946Val	0.019	0.405	9.54E-05	Deleterious	Probably damaging
	rs78190897	*DNAH6*	2	84924823	A/G	Val2550Ala	0.067	-0.220	9.86E-05	Tolerated	Benign
	rs2231926	*EBLN2*	3	73111809	A/G	Ile193Val	0.414	-0.114	9.97E-05	Tolerated	Benign
C16	rs2229291	*CPT2*	1	53676401	A/C	Phe352Cys	0.228	0.162	8.36E-07	Deleterious	Probably damaging
	rs11545725	*LSP1*	11	1902793	T/A	Gln108Leu	0.086	-0.208	1.59E-05	Tolerated	Benign
	rs144196027	*DAG1*	3	49548127	G/C	Leu54Val	0.002	1.343	5.22E-05	Deleterious	Probably damaging
	rs3736946	*ACSL5*	10	114169276	A/G	Met182Val	0.148	-0.155	6.03E-05	Tolerated	Benign
	rs201617733	*KIF20B*	10	91497822	G/A	Ser1035Asn	0.007	-0.674	6.90E-05	Deleterious	Benign
Glycine	rs1047883	*CPS1*	2	211456637	G/A	Thr350Ala	0.470	-0.155	**2.13E-08**	Tolerated	Benign
	rs2278022	*ATMIN*	16	81076821	A/G	Ser240Pro	0.220	-0.130	8.56E-05	Deleterious	Benign
	rs199916502	*EVC*	4	5749917	G/A	Leu328Phe	0.005	-0.748	0.0001	Tolerated	Probably damaging
	rs6784362	*ZPLD1*	3	102157365	T/A	Ile28Phe	0.017	-0.405	0.00012	Tolerated	Benign
	rs6784389	*ZPLD1*	3	102157417	A/G	Asn45Ser	0.017	-0.405	0.00012	Tolerated	Benign
Creatinine	rs8104890	*ZNF614*	19	52519608	G/A	Val415Ile	0.327	-0.107	4.48E-05	Tolerated	Benign
	rs117465739	*ZFR2*	19	3831781	G/A	Ala159Thr	0.034	0.278	4.57E-05	Tolerated	Probably damaging
	rs156697	*GSTO2*	10	106039185	A/G	Asn142Asp	0.251	0.115	7.18E-05	Tolerated	Benign
	rs17200983	*LY6G6F*	6	31675283	C/A	Pro34Gln	0.049	0.228	9.82E-05	Deleterious	Probably damaging
	rs61735710	*SDHAF4*	6	71289188	G/A	Gln46Ter	0.010	0.484	0.000122	NA	NA
lysoPC a C18:2	rs200190874	*L3MBTL4*	18	6241368	A/G	Asn181Asp	0.002	-1.169	6.29E-05	Deleterious	Benign
	rs115407410	*FAM162A*	3	122103120	G/A	Ser3Asn	0.073	0.197	0.00011	Deleterious	Benign
	rs201611745	*FAM69B*	9	139612156	A/G	Val64Ala	0.007	-0.604	0.00012	Tolerated	Benign

	rs139395451	*PPY*	17	42019010	G/A	Arg5Cys	0.001	-1.568	0.00014	Deleterious	NA
	rs4646422	*CYP1A1*	15	75015305	G/A	Gly45Asp	0.191	0.124	0.00032	Deleterious	Possibly damaging
PC aa C34:2	rs72553947	*CMPK1*	1	47838652	A/G	Asn66Ser	0.042	0.268	0.00015	Tolerated	Benign
	rs139694462	*XIRP2*	2	168104747	A/G	Val2282Ala	0.003	0.963	0.00024	Tolerated	NA
	rs115591494	*PRRC2A*	6	31599370	C/G	Glu974Gln	0.002	-1.271	0.00024	NA	NA
	rs1432273	*TTC21B*	2	166797646	A/G	Val201Met	0.215	0.128	0.00024	Deleterious	Probably damaging
	rs189249722	*TNC*	9	117848470	G/A	Gly514Arg	0.001	1.569	0.00034	Deleterious	Probably damaging
PC ae C36:0	rs2108622	*CYP4F2*	19	15990431	G/A	Val433Met	0.325	0.213	**8.19E-13**	Deleterious	Probably damaging
	rs11880184	*OR10H4*	19	16060658	A/G	Thr281Ala	0.125	0.181	1.59E-05	Deleterious	Benign
	rs16980822	*OR10H4*	19	16060248	A/G	His144Arg	0.125	0.180	1.80E-05	Tolerated	Benign
	rs16980994	*OR10H4*	19	16060117	C/A	Asn100Lys	0.125	0.179	1.90E-05	Deleterious	Benign
	rs201354502	*TMEM88*	17	7758532	A/C	Val47Gly	0.003	1.032	1.00E-04	Tolerated	Benign
PC ae C36:2	rs41345745	*MUC2*	11	1081112	C/G	Glu470Gln	0.418	-0.121	4.15E-05	NA	Probably damaging
	rs17646221	*FAM196B*	5	169310213	A/C	Ser230Arg	0.161	0.162	4.36E-05	Tolerated	Benign
	rs74810099	*SNAPC1*	14	62229285	A/C	Met36Arg	0.029	0.318	0.00017	Tolerated	Benign
	rs11541179	*FAM234A*	16	314664	G/A	Arg466Cys	0.003	1.012	0.00019	Deleterious	Possibly damaging
	rs79274660	*FGD2*	6	36982699	A/T	Gln305Leu	0.012	0.470	0.00028	Deleterious	Possibly damaging
Hexose	rs117543061	*BBS9*	7	33427727	G/A	Asp696Asn	0.014	-0.477	2.73E-06	Tolerated	Probably damaging
	rs200511993	*ADAM28*	8	24199172	G/A	Arg578Trp	0.001	-1.591	1.49E-05	Deleterious	Possibly damaging
	rs200200671	*COL6A2*	21	47552333	A/G	Leu976Ser	0.005	-0.642	0.0001	Deleterious	Possibly damaging
	rs116885661	*RPAIN*	17	5329392	C/A	Val139Leu	0.015	0.376	0.0001	Deleterious	Probably damaging
	rs202105093	*ARHGDIG*	16	332719	G/A	Pro195Ser	0.010	-0.458	0.00015	Tolerated	Benign

Chr, chromosome; MAF, minor allele frequency

[*] C14:1, tetradecenoylcarnitine; C16, hexadecanoylcarnitine; lysoPC a C18:2, lysophosphatidylcholine acyl C18:2; PC aa C34:2, phosphatidylcholine diacyl C34:2; PC ae C36:0, phosphatidylcholine acyl-alkyl C36:0; PC ae C36:2, phosphatidylcholine acyl-alkyl C36:2

[†] The allele of each non-synonymous variant is indicated as ‘major allele/minor allele’.

Bold letters indicate the significant associations ($P < 2.05 \times 10^{-7}$).
